# Supplementary material for: Myopericarditis in a Male Adolescent Following the Second Shot of COVID-19 Pfizer m-RNA Vaccine: Typical Example and Analysis of 110 Single Case Reports
Source: Pediatr Rep. 2022 Sep 26;14(4):401–9. doi: 10.3390/pediatric14040048 (PMC9589994; doi:10.3390/pediatric14040048)
Supplement: Supplementary file 1 [file pediatrrep-14-00048-s001.zip › pediatrrep-1879504-supplementary.pdf]

Supplementary Material

Table S1. List of the 110 case reports which have been analysed and extracted information.

Author	Patient's age (years)	Patient's gender	Vaccine type	Vaccine dose	Number of days between vaccine administration and onset of symptoms	Disease	Symptoms	ECG on admission	Troponin	Imaging	Biopsy	Outcome
Walters CG, et al **[43]**	29	Male	Pfizer-BioNTec	Second	4	Myopericarditis	Chest pain	Diffuse ST segment elevation	Increased	Echo (EF 60%)	Not done	Complete resolution
Fritz RO, et al **[44]**	In his 20s	Male	Pfizer-BioNTec	Third	3	Myopericarditis	Chest pain	PR tract sloping and ST elevation in V1-V3	Increased	Echo (depressed EF), c-MRI	Not done	Complete resolution
Ehldouheri E, et al **[45]**	30	Male	Pfizer-BioNTec	Second	7	Myopericarditis	Chest pain, dyspnoea, fever	Diffuse ST segment elevation	Increased	Echo (normal EF)	Not done	Complete resolution
Javed A, et al **[46]**	64	Male	Moderna	Second	7	Pericarditis	Chest pain, dyspnoea	Atrial flutter	Not checked	Echo (normal EF), CT scan	Not done	Complete resolution
Patel K, et al **[47]**	57	Male	Pfizer-BioNTec	Second	7	Pericarditis	Dyspnoea, fever, chills	Normal morphology	Normal	Echo (normal EF), CT scan	Not done	Complete resolution
Generette GS, et al **[48]**	74	Male	Pfizer-BioNTec	Second	14	Myopericarditis	Chest pain, fever	Atrial fibrillation	Normal	Echo (EF not reported)	Not done	Complete resolution
Mizoguchi T, et al **[49]**	88	Female	Pfizer-BioNTec	Second	3	Pericarditis	Chest pain, dyspnoea	PR tract depression	Normal	Echo (normal EF), CT scan	Not done	Complete resolution
Morton Z, et al **[50]**	16	Male	Pfizer-BioNTec	Second	2	Myopericarditis	Chest pain, fever	Diffuse ST segment elevation	Increased	Echo (normal EF)	Not done	Complete resolution
Park S, et al **[51]**	17	Male	Pfizer-BioNTec	First	18	Myocarditis	Chest pain	Diffuse ST segment elevation	Increased	Echo (normal EF), c-MRI	Not done	Complete resolution
Perna F, et al **[52]**	30	Male	Pfizer-BioNTec	Second	5	Pericarditis	Chest pain	Diffuse ST segment elevation	Not measured	Echo (normal EF)	Not done	Complete resolution

								and PR depression				
Sharbatban A, et al **[53]**	25	Male	Moderna	Second	3	Myopericarditis	Chest pain, fatigue	Diffuse ST segment elevation	Increased	Echo (normal EF)	Not done	Complete resolution
Misumi I, et al **[54]**	59	Male	Pfizer-BioNTec	First	7	Pericarditis	Dyspnoea, leg oedema	Poor R wave progression in the precordial leads	Not measured	Echo (normal EF)	Not done	Constrictive pericarditis
Kim SH, et al **[55]**	63	Female	Astrazeneca	Second	3 hours	Myocarditis	Chest pain, dizziness	Abnormal (no other specifications)	Increased	Echo (EF 15%)	Done post-explantation	Heart transplant
Gill J, et al **[56]**	44	Male	Moderna	Second	4	Myopericarditis	Chest pain, dyspnoea	Normal	Increased	Echo (normal EF), c-MRI	Not done	Complete resolution
Fadah K, et al **[57]**	18	Male	Moderna	First	3	Myopericarditis	Chest pain	Diffuse ST segment elevation and PR tract depression	Increased	Echo (borderline EF 50-55%)	Not done	Lost of follow-up
Singh A, et al **[58]**	69	Female	Moderna	Second	1	Pericarditis	Chest pain	Diffuse ST segment elevation	Not tested	Echo (normal EF), CT scan	Not done	Ongoing follow-up
Viani GM, et al **[59]**	59	Male	Pfizer-BioNTec	Second	5	Pericarditis	Chest pain	Normal morphology	Normal	Echo (normal EF), c-MRI	Not done	Ongoing follow-up
Zaki HA, et al **[60]**	55	Female	Pfizer-BioNTec	Third	2	Pericarditis	Fever, chills, dry cough	Normal morphology	Not tested	Echo (normal EF), CT scan	Not done	Complete resolution
Plüß M, et al **[61]**	67	Female	Astrazeneca	First	14	Pericarditis	Fever, weakness, diffuse arthralgia	Not done	Not tested	Transoesophageal echo (normal EF), c-MRI	Not done	Complete resolution
Nakanishi Y, et al **[62]**	70	Female	m-RNA vaccine (no further specifications)	Second	7	Pericarditis	body weight gain, peripheral oedema, dyspnea on effort	Normal morphology	Normal	Echo (normal EF), CT scan	Not done	Complete resolution

Hryniewicki AT, et al **[63]**	18	Male	Pfizer-BioNTec	First	21	Pericarditis	Chest pain, fever, dyspnoea	Diffuse ST segment elevation and PR tract depression	Not tested	Echo (normal EF)	Not done	Ongoing follow-up
Badshah M, et al **[64]**	22	Female	Moderna	Second	1	Myopericarditis	Chest pain	Normal morpholohy	Increased	Echo (EF 48%), c-MRI	Not done	Ongoing follow-up
Ashaari S, et al **[65]**	66	Male	Pfizer-BioNTec	First	7	Pericarditis	Chest pain, dyspnoea	Diffuse ST segment elevation	Increased	ST segment elevation in leads V2-V4	Not done	Complete resolution
Khogali F, et al **[66]**	29	Female	Moderna	Second	10	Perimyocarditis	Fever, fatigue	Diffuse ST segment elevation	Increased	Echo (EF 27%)	Not done	Complete resolution
Horiuchi K, et al **[67]**	32	Male	Moderna	First	5	Myocarditis	Abdominal pain, fever	ST segment elevation in leads D2, aVF, and V1-V3	Increased	Echo (EF 20%)	Yes	Complete resolution
Hoshino N, et al **[68]**	27	Male	Moderna	First	8	Myocarditis	Syncope	Asystole	Not tested	Echo (EF 25%)	Yes	Death
Anthony JA, et al **[69]**	45	Male	Moderna	Second	3	Myocarditis	Chest pain	ST segment elevation in leads V2-V4	Increased	Echo (EF 50%), c-MRI	Not done	Residual chest discomfort and dizziness
Hirsch VG, et al **[70]**	32	Male	Pfizer-BioNTec	First	4	Myocarditis	Shortness of breath	Right bundle branch block	Increased	Echo (EF 47%), c-MRI	Yes	Reduced ventricular systolic function
Etuk AS, et al **[71]**	18	Male	Moderna	First	8 hours	Myocarditis	Chest pain	ST segment elevation in lateral leads D1 and aVL	Increased	Echo (normal EF), c-MRI	Not done	Complete resolution
Alizadeh LS, et al **[72]**	29	Male	Astrazeneca	First	3	Myocarditis	Chest pain, fever	ST segment elevation in postero-lateral leads	Increased	Echo (EF 44%), c-MRI	Not done	Complete resolution

Khan MZ, et al **[73]**	22	Male	Moderna	Second	30	Myocarditis	Abdominal pain	Not reported	Increased	Echo (EF 10%), c-MRI	Not done	Right ventricular dysfunction on milrinone
Kawano H, et al **[74]**	60	Female	Pfizer-BioNTec	Second	24	Myocarditis	Fever	Q waves in inferior leads and V1-V3	Increased	Echo (EF 23%), c-MRI	Yes	Complete resolution
Bae DH, et al **[75]**	38	Female	Moderna	First	4	Myocarditis	Chest pain	ST segment elevation in leads V1-V3	Increased	Echo (normal EF), c-MRI	Yes	Complete resolution
Kimura M, et al **[76]**	69	Male	Pfizer-BioNTec	Not reporter (first?)	7	Myocarditis	Chills, peripheral coldness	ST segment elevation in leads D2, D3, aVF, V1-V4	Increased	Echo (severe global hypokinesia)	Yes	Complete resolution
Marsukjai A, et al **[77]**	55	Female	Astrazeneca	Second	14	Myocarditis	Palpitations	Sinus tachycardia	Increased	Echo (normal EF), c-MRI	Yes	Complete resolution
Ansari U, et al **[78]**	23	Male	Moderna	Second	1	Myocarditis	Chest pain	ST segment elevation in leads D2, D3, aVF	Increased	Echo (moderately reduced EF), c-MRI	Not done	Complete resolution
Asaduzzaman M, et al **[79]**	15	Female	Pfizer-BioNTec	Second	1	Myocarditis	Fever, convulsions	Qtc prolongation	Increased	Echo (EF 46%)	Not done	Complete resolution
Durucan I, et al **[80]**	24	Male	Pfizer-BioNTec	Second	14	Myocarditis	Bilateral lower extremities pain	ST-segment depressions in leads I, II, and aVL	Increased	Echo (EF 46%)	Not done	Complete resolution
Wu CT, et al **[81]**	44	Female	Astrazeneca	First	2	Perimyocarditis	Chest pain, dyspnoea	ST-segment depressions in leadsV1-V2	Increased	Echo (EF 35%), c-MRI	Not done	Complete resolution

Iwamuro A, et al **[82]**	17	Male	Moderna	Second	Same day	Myocarditis	Arthralgia, fever	Diffuse ST segment elevation	Increased	Echo (normal EF), c-MRI	Not done	Complete resolution
Yamamoto S et al **[83]**	13	Male	Pfizer-BioNTec	Second	3	Myocarditis	Chest pain, fever	ST-segment elevation in the II, III, aVf, and V4–6 leads	Increased	Echo (normal EF), c-MRI	Not done	Complete resolution
Olmos CV et al **[84]**	49	Female	Pfizer-BioNTec	Second	6	Myocarditis	Chest discomfort, dyspnoea	Diffuse ST segment elevation and incomplete left bundle branch block	Increased	Echo (normal EF), c-MRI	Yes	AKI, pneumonia, transfer to rehabilitation centre
Olagunjus A et al **[85]**	19	Male	Moderna	First	2	Myocarditis	Chest pain	Diffuse ST segment elevation	Increased	Echo (EF 49%)	Not done	Complete resolution
Dlewati M et al **[86]**	48	Male	Moderna	Second	3	Myocarditis	Fever, fatigue, chills	Normal ECG	Increased	Echo (EF 40%)	Not done	Complete resolution
Mengesha B et al **[87]**	43	Female	Pfizer-BioNTec	Third	2	Myocarditis	Palpitations, shortness of breath	Ventricular tachycardia	Increased	Echo (EF 40-45%), c-MRI	Yes	Defibrillator insertion
Chellapandian SB et al **[88]**	22	Male	Moderna	Second	2	Myocarditis	Chest pain	Normal ECG	Increased	c-MRI	Not done	Complete resolution
Bollano E et al **[89]**	20	Male	Moderna	Second	7	Myocarditis	Chest pain, fever	Normal ECG	Increased	Echo (normal EF), c-MRI	Yes	Chest pain
Hassanzadeh S et al **[90]**	32	Female	Astrazeneca	First	3	Myocarditis	Palpitations, dyspnoea	Normal ECG	Normal	Echo (normal EF)	Not done	Discharged on therapy
Ceylan ME et al **[91]**	57	Female	Pfizer-BioNTec	Second	2	Myocarditis	Vertigo, drop attack	Not reported	Normal	Echo (normal EF)	Not done	Complete resolution
Terán Brage E, et al **[92]**	62	Female	Moderna	Third	1	Myocarditis	Fever, hypotension	Sinus tachycardia	Increased	Echo (severely depressed EF), c-MRI	Not done	Discharged on therapy

Mohammed LM et al **[93]**	19	Male	mRNA vaccine (no further specification)	First	14	Myocarditis	Chest pain	Moderate ST depression in V1-V2	Increased	Echo (normal EF), CT	Not done	Discharged on therapy
Kojima N, at al **[94]**	17	Male	Pfizer-BioNTec	Second	2	Myocarditis	Chest pain, fever, diarrhoea	ST elevation in V2–V5 leads	Increased	Echo (EF 55%), c-MRI	Yes	Complete resolution
Korosoglou G, at al **[95]**	21	Male	Pfizer-BioNTec	Second	2	Myocarditis	Chest pain, fatigue, myalgia, headache	ST elevation in V2–V5 leads	Normal	Echo (EF 45%), c-MRI	Not done	Complete resolution
Mancini N, at al **[96]**	31	Male	Pfizer-BioNTec	Second	4	Myocarditis	Chest pain	ST elevation in V1-V3 leads	Increased	c-MRI	Not done	Complete resolution
Kang DH, at al **[97]**	48	Female	Pfizer-BioNTec	Second	4	Myocarditis	Palpitations	ST elevation in V2–V5 leads	Increased	Echo (EF 15%), c-MRI	Not done	Heart transplantation
Kawakami T, at al **[98]**	45	Female	Moderna	Second	14	Myocarditis	Chest pain	Inverted T waves in inferior leads	Increased	Echo (mildly reduced EF), c-MRI	Not done	Complete resolution
Lee CH at al **[99]**	22	Male	Moderna	Second	5	Myocarditis	Chest pain	ST-segment depression and T-wave inversion in leads V1–V4	Increased	Echo (normal EF), c-MRI	Not done	Complete resolution
Kazama S, at al **[100]**	48	Female	Moderna	Second	7	Myocarditis	Malaise, dyspnoea	ST elevation in V1–V54leads	Increased	Echo (EF 11%), c-MRI	Yes	Complete resolution
Al-Rasbi S, et al **[101]**	37	Male	Pfizer-BioNTec	First	12	Myocarditis	Back pain, left arm pain	Normal morphology	Increased	Echo (EF 45-50%), c-MRI	Not done	Discharged on therapy
Sano M, et al **[102]**	20	Male	Moderna	Second	2	Myocarditis	Chest pain, fever	Diffuse ST segment elevation	Increased	Echo (EF 45-42%), c-MRI	Yes	Complete resolution

Kyaw H, et al **[103]**	24	Male	Pfizer-BioNTec	Second	6 hour	Myocarditis	Chest pain	Normal morphology	Increased	Echo (EF 55%), c-MRI	Not done	Complete resolution
Oka A, et al **[104]**	50	Male	Pfizer-BioNTec	Second	10	Myocarditis	Chest pain, syncope	ST elevation in V1-V4 leads with complete right bundle branch block	Increased	Echo (EF 35%), c-MRI	Yes	Complete resolution
Uesako H, et al **[105]**	26	Male	Pfizer-BioNTec	First	5	Peri-myocarditis	Fatigue, headache, syncope	Ventricular fibrillation	Increased	Echo (mildly reduced EF), c-MRI	Yes	Defibrillator insertion
Fosch X, et al **[106]**	24	Male	Pfizer-BioNTec	Third	1	Myocarditis	Chest pain	diffuse concave ST segment elevation and a PR segment elevation in lead aVR	Increased	Echo (EF 56%), c-MRI	Not done	Discharged on therapy
Naghashzadeh F, et al **[107]**	29	Male	rAd26 and rAd5 vector-based vaccine (Sputnik V)	Second	2	Myocarditis	Chest pain	ST segment elevation in leads V3-V4	Increased	Echo (EF 30-35%), c-MRI	Yes	Complete resolution
Agdamag ACC, et al **[108]**	80	Female	Pfizer-BioNTec	First	7	Myocarditis	Emesis, abdominal discomfort	Non-specific ST-segment and T-wave abnormalities and low voltage QRS complexes throughout	Increased	Echo (normal EF), c-MRI	Yes	Complete resolution
Wong J, et al **[109]**	20	Male	Pfizer-BioNTec	Second	12 hours	Myocarditis	Chest pain, headache	Diffuse ST segment elevation	Increased	Echo (normal EF), c-MRI	Not done	Discharged on therapy

Ohnishi M, et al **[110]**	26	Male	Pfizer-BioNTec	Second	4	Myocarditis	Chest pain	ST segment elevation in I, II, aVL, aVF, V4-V6, and small Q wave in II, III, aVF	Increased	Echo (EF 53%), c-MRI	Not done	Post vaccination syndrome
Aikawa T, et al **[111]**	20	Male	Moderna	Second	2	Myocarditis	Chest pain	ST segment elevation in leads I, aVL, and V2–V6	Increased	Echo (EF not reported), c-MRI	Yes	Complete resolution
Chachar TS, et al **[112]**	24	Male	Pfizer-BioNTec	First	5	Myocarditis	Chest pain	Subtle ST elevation in the precordial leads	Increased	Echo (EF 55%), c-MRI	Not done	Discharged on therapy
Wu B, et al **[113]**	40	Male	Pfizer-BioNTec	First	3	Myocarditis	Chest pain, dyspnoea	Diffuse ST elevation across all leads, except lead III and aVR and V1 where there were ST depressions	Increased	Echo (EF 47%), c-MRI	Yes	Occasional pleuritic, left-sided discomfort
Mimouni H, et al **[114]**	14	Not reported	m-RNA vaccine (no further specification)	Not reported	10	Myocarditis	Extreme asthenia, fever	Sinus tachycardia	Increased	Echo (EF 25%), c-MRI	Not done	Discharged on therapy
Ameratunga R, et al **[115]**	57	Female	Pfizer-BioNTec	First	1	Myocarditis	Fatigue, lethargy	Not done	Not checked	Not done	Yes	Death
Umei TC, et al **[116]**	20	Male	Moderna	Second	2	Myopericarditis	Chest pain, fever	Diffuse ST elevation	Increased	Echo (EF 55%), c-MRI	Not done	Complete resolution

Nagasaka T, et al **[117]**	23	Male	Pfizer-BioNTec	Second	3	Myocarditis	Chest pain, fever	Subtle ST elevation	Increased	Echo (EF not reported), c-MRI	Yes	Complete resolution
Tinoco M, et al **[118]**	39	Male	Pfizer-BioNTec	Second	3	Perimyocarditis	Chest pain, fever	ST segment elevation with depressed PR segment in leads V3 to V6, DI, DII, aVL, and ST segment depression with PR segment elevation in lead aVR	Increased	Echo (EF 48%), c-MRI	Not done	Discharged on therapy
Maeda M, et al **[119]**	29	Male	Moderna	First	4	Myocarditis	Chest pain	ST-T changes in D3, V1, aVL	Increased	c-MRI	Not done	Not reported
Kadwalwala M, et al **[120]**	38	Female	Moderna	First	8	Myopericarditis	Chest pain, shortness of breath	Right bundle branch block	Increased	Echo (EF 30-10%), c-MRI	Yes	Discharged on therapy. Reduced EF
Miqdad MA, et al **[121]**	18	Male	Pfizer-BioNTec	Second	4	Myocarditis	Chest pain	ST segment elevation in leads I, II along with V2-V6	Increased	Echo (EF 63%), c-MRI	Not done	Complete resolution
Lim Y, et al **[122]**	38	Female	Pfizer-BioNTec	Not reported	8	Myocarditis	Chest pain	Diffuse ST segment elevation	Increased	Echo (EF 10%), c-MRI	Yes	Complete resolution
Azir M, et al **[123]**	17	Male	Pfizer-BioNTec	Second	3	Myocarditis	Chest pain, dyspnoea	Lateral ST elevation	Increased	Echo (EF 55%), c-MRI	Not done	Complete resolution
Kaneta K, et al **[124]**	25	Male	Moderna	Second	3	Myocarditis	Chest pain, fever	St elevation in inferior and lateral leads	Increased	Echo (EF not reported), c-MRI	Not done	Complete resolution

Schmitt P, et al **[125]**	19	Male	Pfizer-BioNTec	Second	3	Myocarditis	Chest pain	Diffuse ST segment elevation and PR depression in inferior leads	Increased	Echo (EF 72%), c-MRI	Not done	Complete resolution
Habedank D, et al **[126]**	60	Male	Moderna	Second	3	Myocarditis	Syncope	Normal morphology	Increased	Echo (normal EF), c-MRI	Not done	Complete resolution
Facetti S, et al **[127]**	20	Male	Pfizer-BioNTec	Second	2	Myopericarditis	Chest pain	St segment elevation in inferior leads and V2-V4	Increased	Echo (normal EF), c-MRI	Not done	Complete resolution
Riedel PG, et al **[128]**	47	Male	Inactivated vaccine (Sinovac)	Second	Not specified	Myocarditis	Chest pain	Left ventricular overload	Increased	Echo (EF 15%), c-MRI	Not done	Persisting fatigue and dyspnoea
Choi S, et al **[129]**	22	Male	Pfizer-BioNTec	First	5	Myocarditis	Syncope	Ventricular fibrilation	Not tested	Not done	Done with authopsy	Death
Gautam N, et al **[130]**	66	Male	Pfizer-BioNTec	Second	90	Myocarditis	Chest pain	ST elevation in anterior leads	Increased	c-MRI	Not done	Complete resolution
Kim D, et al **[131]**	29	Male	Pfizer-BioNTec	Second	1	Myopericarditis	Chest pain	Normal morphology	Increased	Echo (EF 62%), c-MRI	Not done	Complete resolution
Sakaguchi S, et al **[132]**	49	Male	Pfizer-BioNTec	Second	11	Myocarditis	Fever, cough	Low voltage in the limb leads and poor R waves in V1–2	Increased	Echo (EF 33%), c-MRI	Yes	Complete resolution
Nguyen TD, et al **[133]**	20	Male	Moderna	First	12 hours	Myocarditis	Chest pain, fever	Pre-excitation features	Increased	c-MRI	Yes	Complete resolution
Tailor PD, et al **[134]**	44	Male	Moderna	Second	4	Myocarditis	Chest pain	ST-segment elevation in the lateral limb and	Increased	Echo (EF 40%), c-MRI	Not done	Complete resolution

								precordial leads				
Ehrlic P, et al **[25]**	40	Male	Pfizer-BioNTec	First	6	Myocarditis	Chest pain, fever, shortness of breath	Normal morphology	Increased	Echo (EF 45%)	Yes	Complete resolution
Sulemankhil I, et al **[135]**	33	Male	Janssen	First	2	Myocarditis	Chest pain	Normal morphology	Increased	Echo (normal EF), c-MRI	Not done	Discharged on therapy
Kim IC, et al **[136]**	24	Male	Pfizer-BioNTec	Second	1	Myocarditis	Chest pain	Diffuse ST elevation in lead II, and V2-V5	Increased	Echo (normal EF), c-MRI	Not done	Complete resolution
Patrignani A, et al **[137]**	56	Male	Pfizer-BioNTec	First	4	Myocarditis	Epigastric pain	Normal morphology	Increased	Echo (normal EF), c-MRI	Not done	Complete resolution
Hasnie AA, et al **[138]**	22	Male	Moderna	First	3	Perimyocarditis	Chest pain	Diffuse ST segment elevation	Increased	Echo EF (50-55%), c-MRI	Not done	Discharged on therapy
Isaak A, et al **[139]**	15	Male	Pfizer-BioNTec	Second	1	Myocarditis	Fever, myalgia, intermittent tachycardia	ST-segment elevation in the left precordial leads	Increased	Echo (normal EF), c-MRI	Not done	Discharged (on therapy?)
Williams CB, et al **[140]**	34	Male	Moderna	Second	3	Perimyocarditis	Chest pain, fever, myalgia	Lateral PR-segment depression and ST-segment elevation mirrored in the aVR with PR-segment elevation and ST-segment depression	Increased	Echo (EF 43%), c-MRI	Not done	Discharged on therapy
Nassar M, et al **[141]**	70	Female	Janssen	First	2	Myocarditis	Dyspnoea	T wave inversions in leads V4-V6 without	Normal	Echo (EF 10%)	Not done	Death

								any ST-segment change				
Cereda A, et al **[142]**	21	Male	Pfizer-BioNTec	Second	2	Myocarditis	Chest pain, fever	Diffuse St segment elevation	Increased	Echo (normal EF), c-MRI	Not done	Complete resolution
Watkins K, et al **[143]**	20	Male	Pfizer-BioNTec	Second	2	Myocarditis	Chest pain	Diffuse St segment elevation	Increased	Echo (c-MRI 59%), c-MRI	Not done	Discharged on therapy
Deb A, et al **[144]**	67	Male	Moderna	Second	6 hours	Myocarditis	Dyspnoea, fever	Non specific ST/T wave changes	Increased	Echo (EF 50-54%)	Not done	Complete resolution
Habib MB, et al **[145]**	37	Male	Pfizer-BioNTec	Second	3	Myocarditis	Chest pain	ST segment elevation in anterior leads	Increased	Echo (EF 57%), c-MRI	Not done	Complete resolution
Minocha PK, et al **[146]**	17	Male	Pfizer-BioNTec	Second	1	Myocarditis	Body aches, fever	Diffuse St segment elevation	Increased	Echo (EF 53%)	Not done	Complete resolution
Muthukumar A, et al **[147]**	52	Male	Moderna	Second	3	Myocarditis	Chest pain, headache	Incomplete right bundle branch block	Increased	Echo (normal EF), c-MRI	Not done	Discharged on therapy
McLean K, et al **[148]**	16	Male	Pfizer-BioNTec	Second		Myopericarditis	Chest pain	ST elevation in V2-V6 and I and aVL	Increased	Echo (normal EF), c-MRI	Not done	Discharged on medical therapy
D'Angelo T, et al **[23]**	30	Male	Pfizer-BioNTec	Second	3	Myocarditis	Chest pain, dyspnoea	Subtle St segment elevation in V2-V4	Increased	Echo (normal EF), c-MRI	Not done	Complete resolution
Albert E, et al **[149]**	24	Male	Moderna	Second	4	Myocarditis	Chest pain	Normal morphology	Increased	Echo (EF 65%), c-MRI	Not done	Discharged on therapy
Bautista García J, et al **[150]**	39	Male	Pfizer-BioNTec	Second	6 hours	Myocarditis	Chest pain, fever	Diffuse ST segment elevation	Increased	c-MRI	Not done	Complete resolution

References

43. Walters, C.G.; Jaiswal, D.D.; Hu, T.X.; Kim, S.S. Myopericarditis in a Young Adult Secondary to COVID-19 Vaccination. Methodist Debakey Cardiovasc. J. 2021, 17, 13–17.
44. Fritz, R.O.; Betageri, O.; Chakrala, T.; Kim, J.; Al-Ani, M.; Omar, A. Severe myopericarditis following the third dose of an mRNA COVID-19 vaccine: Utility of a multimodal treatment approach. BMJ Case Rep. 2022, 15, e249533.
45. Elhouderi, E.; Elsawalhy, E.; Kabbani, M. Myopericarditis after mRNA COVID-19 Vaccine in a Patient with Recent History of COVID-19. Cureus 2022, 14, e25264.
46. Javed, A.; Medina, Y.; Tian, J.; Alvi, M.J.; Sahra, S.; Rojas-Marte, G. An Unusual Case of Hemorrhagic Pleuropericarditis after COVID-19 Vaccination. Cureus 2022, 14, e24828.
47. Patel, K.; Dalal, S.; Tran, D.; Patel, A.; McMackin, C. COVID-19 mRNA Vaccine Induced Pericarditis with Large Pericardial Effusion Followed by New-Onset Arrhythmia. Cureus 2022, 14, e24699.
48. Generette, G.S.; Troyer, J.; Hemenway, A.; Al Zoubi, M. Myopericarditis with hemorrhagic pericardial effusion following BNT162b2 mRNA COVID-19 vaccine. IDCases 2022, 28, e01511.
49. Mizoguchi, T.; Yokoi, M.; Shintani, Y.; Yamamoto, J.; Mori, K.; Fujita, H.; Ito, T.; Sugiura, T.; Seo, Y. A case of an elderly female who developed subacute pleuropericarditis following BNT162b2 mRNA COVID-19 vaccination. J. Cardiol. Cases 2022, 26, 225–228. https://doi.org/10.1016/j.jccase.2022.04.020.
50. Morton, Z.; Green, D.; Grisham, M. A case of myopericarditis following administration of the Pfizer COVID-19 vaccine. Arch. Clin. Cases 2022, 9, 1–5.
51. Park, S.; You, J. A Case of Myocarditis Presenting with a Hyperechoic Nodule after the First Dose of COVID-19 mRNA Vaccine. J. Korean Med. Sci. 2022, 37, e131.
52. Perna, F.; Verecchia, E.; Pinnacchio, G.; Gerardino, L.; Brucato, A.; Manna, R. Rapid resolution of severe pericardial effusion using anakinra in a patient with COVID-19 vaccine-related acute pericarditis relapse: A case report. Eur. Heart. J. Case Rep. 2022, 6, ytac123.
53. Sharbatdaran, A.; Chahal, Y.; Molaei, M.; Bhavsar, D. A rare case of COVID-19 vaccine-induced myopericarditis in a young adult. Radiol. Case Rep. 2022, 17, 1916–1920.
54. Misumi, I.; Ogata, A.; Fukuda, K.; Sato, K.; Nagano, M.; Usuku, H.; Tsujita, K. Constrictive pericarditis following mRNA COVID-19 vaccination in a patient with systemic sclerosis. J. Cardiol. Cases 2022, 26, 97–100.
55. Kim, S.H.; Lee, S.Y.; Kim, G.Y.; Oh, J.S.; Kim, J.; Chun, K.J.; Ju, M.H.; Lee, C.H.; Song, Y.J.; Na, J.Y. A Case of Heart Trans-plantation for Fulminant Myocarditis after ChAdOx1 nCoV-19 Vaccination. J. Korean Med. Sci. 2022, 37, e104.
56. Gill, J.; Mallari, A.J.P.; Zahra, F. Transient Myopericarditis Following Vaccination for COVID-19. J. Med. Cases 2022, 13, 80–84.
57. Fadah, K.; Abolbashari, M.; Ojha, C.; Alkhateeb, H. Acute Myopericarditis after First Dose of mRNA-1273 SARS-CoV-2 Vaccine in a Young Adult. Cureus 2022, 14, e22111.
58. Singh, A.; Nguyen, L.; Everest, S.; Afzal, S.; Shim, A. Acute Pericarditis Post mRNA-1273 COVID Vaccine Booster. Cureus 2022, 14, e22148.
59. Viani, G.M.; Pedrotti, P.; Seregni, R.; Antonio, B. Effusive-constrictive pericarditis after the second dose of BNT162b2 vaccine (Comirnaty): A case report. Eur. Heart J. Case Rep. 2022, 6, ytac012.
60. Zaki, H.A.; Zahran, A.; Abdelrahim, M.; Elnabawy, W.A.; Kaber, Y. A Case of Acute Viral Pericarditis Complicated with Pericardial Effusion Induced by Third Dose of COVID Vaccination. Cureus 2022, 14, e21207.
61. Plüß, M.; Mese, K.; Kowallick, J.T.; Schuster, A.; Tampe, D.; Tampe, B. Case Report: Cytomegalovirus Reactivation and Peri-carditis Following ChAdOx1 nCoV-19 Vaccination Against SARS-CoV-2. Front. Immunol. 2022, 12, 784145.
62. Nakanishi, Y.; Honda, S.; Yamano, M.; Kawasaki, T.; Yoshioka, K. Constrictive pericarditis after SARS-CoV-2 vaccination: A case report. Int. J. Infect. Dis. 2022, 116, 238–240.
63. Hryniewicki, A.T.; Tolia, V.M.; Nene, R.V. Cardiac Tamponade after COVID-19 Vaccination. J. Emerg. Med. 2022, 62, 250–253.
64. Badshah, M.; Shriver, J.; Rynders, B.; Sjovold, A.; Shaukat, M.H.S.; Rajpurohit, N. MODERNA mRNA-1273 vaccine-associated myopericarditis in a patient with a subclinical autoimmune predisposition. J. Cardiol. Cases 2021, 24, 227–229.
65. Ashaari, S.; Sohaib, H.A.; Bolger, K. A case report: Symptomatic pericarditis post-COVID-19 vaccination. Eur. Heart J. Case Rep. 2021, 5, ytab375.
66. Khogali, F.; Abdelrahman, R. Unusual Presentation of Acute Perimyocarditis Following SARS-COV-2 mRNA-1237 Moderna Vaccination. Cureus 2021, 13, e16590.
67. Horiuchi, K.; Kosugi, S.; Abe, H.; Ueda. Y. Fulminant myocarditis after the first dose of mRNA-1273 vaccination in a patient with previous COVID-19: A case report. Eur. Heart J. Case Rep. 2022, 6, ytac290.
68. Hoshino, N.; Yanase, M.; Ichiyasu, T.; Kuwahara, K.; Kawai, H.; Muramatsu, T.; Ishii, H.; Tsukamoto, T.; Morimoto, S.I.; Izawa, H. An autopsy case report of fulminant myocarditis: Following mRNA COVID-19 vaccination. J. Cardiol. Cases 2022, Epub ahead of print. https://doi.org/10.1016/j.jccase.2022.06.006.
69. Anthony, J.A.; Echeverry, T.; Fishberg, R.D. The Rise of mRNA COVID-19 Vaccine-Associated Myocarditis and Its Implications on the Future Use of This New Vaccine Platform. Cureus 2022, 14, e25631.

70. Hirsch, V.G.; Schallhorn, S.; Zwadlo, C.; Diekmann, J.; Länger, F.; Jonigk, D.D.; Kempf, T.; Schultheiss, H.P.; Bauersachs, J. Giant cell myocarditis after first dose of BNT162b2—A case report. Eur. J. Heart Fail. 2022, 24, 1319–1322.
71. Etuk, A.S.; Jackson, I.N.; Panayiotou, H. A Rare Case of Myocarditis after the First Dose of Moderna Vaccine in a Patient with Two Previous COVID-19 Infections. Cureus 2022, 14, e24802.
72. Alizadeh, L.S.; Koch, V.; Yel, I.; Grünewald, L.D.; Mathies, D.; Martin, S.; Vogl, T.J.; Rauschning, D.; Booz, C. A case of myo-carditis after COVID-19 vaccination: Incidental or consequential? Heliyon 2022, 8, e09537.
73. Khan, M.Z.; Janus, S.; Franklin, S.; Figueredo, V.; Baqi, A.; Alvarez, R. COVID-19 Vaccination-Induced Cardiomyopathy Re-quiring Permanent Left Ventricular Assist Device. Cureus 2022, 14, e24477.
74. Kawano, H.; Motokawa, T.; Kurohama, H.; Okano, S.; Akashi, R.; Yonekura, T.; Ikeda, S.; Izumikawa, K.; Maemura, K. Ful-minant Myocarditis 24 Days after Coronavirus Disease Messenger Ribonucleic Acid Vaccination. Intern. Med. 2022, 61, 2319–2325.
75. Bae, D.H.; Kim, M.; Lee, D.I.; Lee, J.H.; Kim, S.; Lee, S.Y.; Bae, J.W.; Hwang, K.K.; Kim, D.W.; Cho, M.C. Simultaneous Occurrence of Immune-Mediated Thrombocytopenia and Myocarditis after mRNA-1273 COVID-19 Vaccination: A Case Report. J. Korean Med. Sci. 2022, 37, e169.
76. Kimura, M.; Hashimoto, T.; Noda, E.; Ishikawa, Y.; Ishikita, A.; Fujino, T.; Matsushima, S.; Ide, T.; Kinugawa, S.; Nagaoka, K.; et al. Fulminant necrotizing eosinophilic myocarditis after COVID-19 vaccination survived with mechanical circulatory support. ESC Heart Fail. 2022, 9, 2732–2737.
77. Marsukjai, A.; Theerasuwipakorn, N.; Tumkosit, M.; Chattranukulchai, P.; Srichomkwun, P.; Prechawat, S. Concomitant myocarditis and painless thyroiditis after AstraZeneca coronavirus disease 2019 vaccination: A case report. J. Med. Case Rep. 2022, 16, 212.
78. Ansari, U.; Britsch, S.; Rogowski, S.; Duerschmied, D.; Papavassiliu, T. Case Report: Transient Increase of CMR T1 Mapping Indices in a Patient with COVID-19 mRNA Vaccine Induced Acute Myocarditis. Front. Cardiovasc. Med. 2022, 9, 880717.
79. Asaduzzaman, M.; Purkayastha, B.; Alam, M.M.J.; Chakraborty, S.R.; Roy, S.; Ahmed, N. COVID-19 mRNA vaccine-associated encephalopathy, myocarditis, and thrombocytopenia with excellent response to methylprednisolone: A case report. J. Neu-roimmunol. 2022, 368, 577883.
80. Durucan, I.; Guner, S.; Kilickiran Avci, B.; Unverengil, G.; Melikoglu, M.; Ugurlu, S. Post Covıd-19 Vaccınatıon Inflammatory Syndrome: A Case Report. Mod. Rheumatol. Case Rep. 2022, 378–406. https://doi.org/10.1093/mrcr/rxac041.
81. Wu, C.T.; Chin, S.C.; Chu, P.H. Acute Fulminant Myocarditis after ChAdOx1 nCoV-19 Vaccine: A Case Report and Literature Review. Front. Cardiovasc. Med. 2022, 9, 856991.
82. Iwamuro, A.; Sasa, T.; Kawai, T.; Taguchi, M.; Izuhara, M.; Uegaito, T.; Shioji, K. A 17-year-old male with acute myocarditis following mRNA-1273 vaccination in Japan. J. Cardiol. Cases 2022, 26, 108–110.
83. Yamamoto, S.; Arita, Y.; Ogasawara, N. Myocarditis Following the Second Dose of COVID-19 Vaccination in a Japanese Ad-olescent. Cureus 2022, 14, e23474.
84. Olmos, C.V.; Trahan, S.; Rochon, A.; Ducharme, A. Severe myocarditis after SARS-CoV-2 vaccination in a 49-year-old woman. CMAJ 2022, 194, E581–E584.
85. Olagunju, A.; Moradi, A.; Johnson, B.; Lebaron, Z.; Johnson, R.; Mehdizadeh, A. Acute Myocarditis Following Vaccination with the First Dose of the mRNA-1273 Vaccine. J. Investig. Med. High Impact Case Rep. 2022, 10, 23247096221092291.
86. Dlewati, M.; Park, K.; Rawat, S.; Conte, J.; Bhadha, K. COVID-19 mRNA Vaccine-Associated Myocarditis Presenting as STEMI in a 48-Year-Old Male. Case Rep. Cardiol. 2022, 2022, 2284530.
87. Mengesha, B.; Asenov, A.G.; Hirsh-Raccah, B.; Amir, O.; Pappo, O.; Asleh, R. Severe Acute Myocarditis after the Third (Booster) Dose of mRNA COVID-19 Vaccination. Vaccines 2022, 10, 575.
88. Chellapandian, S.B.; Turkmen, S.; Salim, I.; Chinnakaruppan, S.; Mohammad, J. Myocarditis following COVID-19 mRNA (mRNA-1273) vaccination. Clin. Case Rep. 2022, 10, e05741.
89. Bollano, E.; Bergh, N.; Dudás, A.; Bobbio, E.; Polte, C.L. Somatostatin receptor positron emission tomography/computed tomography in myocarditis following mRNA COVID-19 vaccination. Eur. Heart J. Case Rep. 2022, 6, ytac117.
90. Hassanzadeh, S.; Sadeghi, S.; Mirdamadi, A.; Nematollahi, A. Myocarditis following AstraZeneca (an adenovirus vector vaccine) COVID-19 vaccination: A case report. Clin. Case Rep. 2022, 10, e05744.
91. Ceylan, M.E.; Önen Ünsalver, B.; Dönmez, A.; Kaya Yertutanol, F.D.; Evrensel, A.; Ceylan, H.Z. A case of myocarditis and isolated hypopotassemia after Biontech-Pfizer vaccine for Covid-19. Vaccine 2022, 40, 2897–2898.
92. Terán Brage, E.; Roldán Ruíz, J.; González Martín, J.; Oviedo Rodríguez, J.D.; Vidal Tocino, R.; Rodríguez Diego, S.; Sánchez Hernández, P.L.; Bellido Hernández, L.; Fonseca Sánchez, E. Fulminant myocarditis in a patient with a lung adenocarcinoma after the third dose of modern COVID-19 vaccine. A case report and literature review. Curr. Probl. Cancer Case Rep. 2022, 6, 100153.
93. Mohammed, L.M.; Dhillon, V.; Bong, J.P.; Patri, J. Myocarditis Secondary to COVID-19 mRNA Vaccine: A Case Report. Cureus 2022, 14, e22345.

94. Kojima, N.; Tada, H.; Okada, H.; Yoshida, S.; Sakata, K.; Usui, S.; Ikeda, H.; Okajima, M.; Kawashiri, M.A.; Takamura, M. Case Report: Myocarditis Associated with COVID-19 mRNA Vaccination Following Myocarditis Associated with Campylobacter Jejuni. Front. Cardiovasc. Med. 2022, 9, 837759.
95. Korosoglou, G.; Nunninger, P.; Giusca, S. Case Report: Disappearance of Late Gadolinium Enhancement and Full Functional Recovery in a Young Patient with SARS-CoV-2 Vaccine-Related Myocarditis. Front. Cardiovasc. Med. 2022, 9, 852931.
96. Mancini, N.; Cortigiani, L.; Aquaro, G.; Bovenzi, F.M. Raro caso di miocardite ed embolia polmonare dopo vaccino a mRNA BNT162b2 [A rare case of myocarditis and pulmonary embolism after BNT162b2 mRNA vaccine]. G. Ital. Cardiol. 2022, 23, 244–246.
97. Kang, D.H.; Na, J.Y.; Yang, J.H.; Moon, S.H.; Kim, S.H.; Jung, J.J.; Cha, H.J.; Ahn, J.H.; Park, Y.W.; Cho, S.Y.; et al. Fulminant Giant Cell Myocarditis following Heterologous Vaccination of ChAdOx1 nCoV-19 and Pfizer-BioNTech COVID-19. Medicina 2022, 58, 449.
98. Kawakami, T.; Yahagi, K.; Sekiguchi, M.; Ishizawa, T.; Nonaka, H.; Setoguchi, N.; Watanabe, Y.; Nakase, M.; Horiuchi, Y.; Asami, M.; et al. Acute Myocarditis in a Patient Following mRNA-1273 SARS-CoV-2 Vaccination. Intern. Med. 2022, 61, 1371–1374.
99. Lee, C.H.; Kong, E.J. FDG PET/MRI of Acute Myocarditis after mRNA COVID-19 Vaccination. Clin. Nucl. Med. 2022, 47, e421–e422.
100. Kazama, S.; Okumura, T.; Kimura, Y.; Ito, R.; Araki, T.; Mizutani, T.; Oishi, H.; Kuwayama, T.; Hiraiwa, H.; Kondo, T.; et al. Biopsy-Proven Fulminant Myocarditis Requiring Mechanical Circulatory Support Following COVID-19 mRNA Vaccination. CJC Open 2022, 4, 501–505.
101. Al-Rasbi, S.; Al-Maqbali, J.S.; Al-Farsi, R.; Al Shukaili, M.A.; Al-Riyami, M.H.; Al Falahi, Z.; Al Farhan, H.; Al Alawi, A.M. Myocarditis, Pulmonary Hemorrhage, and Extensive Myositis with Rhabdomyolysis 12 Days after First Dose of Pfiz-er-BioNTech BNT162b2 mRNA COVID-19 Vaccine: A Case Report. Am. J. Case Rep. 2022, 23, e934399.
102. Sano, M.; Murai, R.; Kim, K.; Furukawa, Y. Cardiac magnetic resonance findings in acute myocarditis after mRNA COVID-19 vaccination. J. Cardiol. Cases 2022, 26, 17–20.
103. Kyaw, H.; Shajahan, S.; Gulati, A.; Synn, S.; Khurana, S.; Nazar, N.; Shrestha, S.; Kerstein, J. COVID-19 mRNA Vac-cine-Associated Myocarditis. Cureus 2022, 14, e21009.
104. Oka, A.; Sudo, Y.; Miyoshi, T.; Ozaki, M.; Kimura, Y.; Takagi, W.; Ugawa, S.; Okada, T.; Nosaka, K.; Doi, M. Fulminant myo-carditis after the second dose of COVID-19 mRNA vaccination. Clin. Case Rep. 2022, 10, e05378.
105. Uesako, H.; Fujikawa, H.; Hashimoto, S.; Wakabayashi, T. Prominent J Waves and Ventricular Fibrillation Caused by Myo-carditis and Pericarditis after BNT162b2 mRNA COVID-19 Vaccination. Can. J. Cardiol. 2022, 38, 844–847.
106. Fosch, X.; Serra, J.; Torres, P.L.; Preda, L.; González, R.; Mojer, F. Miocarditis aguda tras la tercera dosis de la vacuna BNT162b2 contra la COVID-19 [Acute myocarditis after a third dose of the BNT162b2 COVID-19 vaccine]. Rev. Esp. Cardiol. 2022.
107. Naghashzadeh, F.; Shafaghi, S.; Dorudinia, A.; Naji, S.A.; Marjani, M.; Amin, A.; Mohamadifar, A.; Noorali, S.; Kashani, B.S. Myocarditis following rAd26 and rAd5 vector-based COVID-19 vaccine: Case report. ESC Heart Fail. 2022, 9, 1483–1486.
108. Agdamag, A.C.C.; Gonzalez, D.; Carlson, K.; Konety, S.; McDonald, W.C.; Martin, C.M.; Maharaj, V.; Alexy, T. Fulminant myocarditis following coronavirus disease 2019 vaccination: A case report. Eur. Heart J. Case Rep. 2022, 6, ytac007.
109. Wong, J.; Sharma, S.; Yao, J.V.; Aggarwal, A.; Grigg, L. COVID-19 mRNA vaccine (Comirnaty)-induced myocarditis. Med. J. Aust. 2022, 216, 122–123.
110. Ohnishi, M.; Tanaka, Y.; Nishida, S.; Sugimoto, T. Case report of acute myocarditis after administration of coronavirus disease 2019 vaccine in Japan. Eur. Heart J. Case Rep. 2022, 6, ytab534.
111. Aokawa, T.; Ogino, J.; Kita, Y.; Funayama, N.; Oyama-Manabe, N. Non-infectious endocarditis and myocarditis after COVID-19 mRNA vaccination. Eur. Heart J. Case Rep. 2022, 6, ytab533.
112. Chachar, T.S.; Yousuf, N.; Sulaibikh, L.; Abdulqader, F.; Alqahtani, M. First Report of Acute Myocarditis Post-Pfizer-BioNTech COVID-19 Vaccination in the Kingdom of Bahrain. Cureus 2021, 13, e20313.
113. Wu, B.; Mittal, N.; Adler, E.D.; Hong, K.N. Acute myocarditis after receiving first dose of BNT162b2 mRNA vaccine. J. Cardiol. Cases 2022, 25, 348–350.
114. Mimouni, H.; Bahouh, C.; Amaqdouf, S.; Laaribi, I.; Baddi, M.; Berichi, S.; Bkiyar, H.; Ismaili, N.; El Ouafi, N.; Housni, B. Cardiogenic shock revealing myocarditis after mRNA vaccination against covid-19: Case report and brief review for the first case in Morocco. Ann. Med. Surg. 2022, 74, 103210.
115. Ameratunga, R.; Woon, S.T.; Sheppard, M.N.; Garland, J.; Ondruschka, B.; Wong, C.X.; Stewart, R.A.H.; Tatley, M.; Stables, S.R.; Tse, R.D. First Identified Case of Fatal Fulminant Necrotizing Eosinophilic Myocarditis Following the Initial Dose of the Pfizer-BioNTech mRNA COVID-19 Vaccine (BNT162b2, Comirnaty): An Extremely Rare Idiosyncratic Hypersensitivity Re-action. J. Clin. Immunol. 2022, 42, 441–447.
116. Umei, T.C.; Kishino, Y.; Watanabe, K.; Shiraishi, Y.; Inohara, T.; Yuasa, S.; Fukuda, K. Recurrence of Myopericarditis Following mRNA COVID-19 Vaccination in a Male Adolescent. CJC Open 2022, 4, 350–352.
117. Nagasaka, T.; Koitabashi, N.; Ishibashi, Y.; Aihara, K.; Takama, N.; Ohyama, Y.; Yokoyama, T.; Kaneko, Y. Acute myocarditis associated with COVID-19 vaccination: A case report. J. Cardiol. Cases 2022, 25, 285–288.

118. Tinoco, M.; Leite, S.; Faria, B.; Cardoso, S.; Von Hafe, P.; Dias, G.; Cardoso, F.; Pereira, T.; Machado, I.; Lourenço, A. Peri-myocarditis Following COVID-19 Vaccination. Clin. Med. Insights Cardiol. 2021, 15, 11795468211056634.
119. Maeda, M.; Isawa, T.; Tada, N. Definite Acute Myocarditis after Coronavirus Disease 2019 mRNA Vaccination. Circ. J. 2022, 86, 724.
120. Kadwalwala, M.; Chadha, B.; Ortoleva, J.; Joyce, M. Multimodality imaging and histopathology in a young man presenting with fulminant lymphocytic myocarditis and cardiogenic shock after mRNA-1273 vaccination. BMJ Case Rep. 2021, 14, e246059.
121. Miqdad, M.A.; Nasser, H.; Alshehri, A.; Mourad, A.R. Acute Myocarditis Following the Administration of the Second BNT162b2 COVID-19 Vaccine Dose. Cureus 2021, 13, e18880.
122. Lim, Y.; Kim, M.C.; Kim, K.H.; Jeong, I.S.; Cho, Y.S.; Choi, Y.D.; Lee, J.E. Case Report: Acute Fulminant Myocarditis and Cardiogenic Shock after Messenger RNA Coronavirus Disease 2019 Vaccination Requiring Extracorporeal Cardiopulmonary Resuscitation. Front. Cardiovasc. Med. 2021, 8, 758996.
123. Azir, M.; Inman, B.; Webb, J.; Tannenbaum, L. STEMI Mimic: Focal Myocarditis in an Adolescent Patient after mRNA COVID-19 Vaccine. J. Emerg. Med. 2021, 61, e129–e132.
124. Kaneta, K.; Yokoi, K.; Jojima, K.; Kotooka, N.; Node, K. Young Male with Myocarditis Following mRNA-1273 Vaccination Against Coronavirus Disease-2019 (COVID-19). Circ. J. 2022, 86, 721.
125. Schmitt, P.; Demoulin, R.; Poyet, R.; Capilla, E.; Rohel, G.; Pons, F.; Jégo, C.; Sidibe, S.; Druelle, A.; Brocq, F.X.; et al. Acute Myocarditis after COVID-19 vaccination: A case report. Rev. Med. Interne 2021, 42, 797–800.
126. Habedank, D.; Lagast, A.; Novoa-Usme, M.; Atmowihardjo, I. A case of myocarditis in a 60-year-old man 48 h after mRNA vaccination against SARS-CoV2. Clin. Res. Cardiol. 2022, 111, 230–232.
127. Facetti, S.; Giraldi, M.; Vecchi, A.L.; Rogiani, S.; Nassiacos, D. Miocardite acuta in giovane adulto due giorni dopo vaccino Pfizer [Acute myocarditis in a young adult two days after Pfizer vaccination]. G. Ital. Cardiol. 2021, 22, 891–893.
128. Riedel, P.G.; Sakai, V.F.; Toniasso, S.C.C.; Brum, M.C.B.; Fernandes, F.S.; Pereira, R.M.; Baldin, C.P.; Baldin, C.C.; Takahasi, A.Y.; Shiki, H.; et al. Heart failure secondary to myocarditis after SARS-CoV-2 reinfection: A case report. Int. J. Infect. Dis. 2021, 113, 175–177.
129. Choi, S.; Lee, S.; Seo, J.W.; Kim, M.J.; Jeon, Y.H.; Park, J.H.; Lee, J.K.; Yeo, N.S. Myocarditis-induced Sudden Death after BNT162b2 mRNA COVID-19 Vaccination in Korea: Case Report Focusing on Histopathological Findings. J. Korean Med. Sci. 2021, 36, e286.
130. Gautam, N.; Saluja, P.; Fudim, M.; Jambhekar, K.; Pandey, T.; Al'Aref, S. A Late Presentation of COVID-19 Vaccine-Induced Myocarditis. Cureus 2021, 13, e17890.
131. Kim, D.; Choi, J.H.; Jang, J.Y.; So, O.; Cho, E.; Choi, H.; Hong, K.S.; Park, K.T. A Case Report for Myopericarditis after BNT162b2 COVID-19 mRNA Vaccination in a Korean Young Male. J. Korean Med. Sci. 2021, 36, e277.
132. Sakaguchi, S.; Fujimoto, N.; Ichikawa, K.; Izumi, D.; Katsuta, K.; Takafuji, M.; Imanaka-Yoshida, K.; Dohi, K. Myopericarditis after COVID-19 mRNA Vaccination. Circ. J. 2022, 86, 472.
133. Nguyen, T.D.; Mall, G.; Westphal, J.G.; Weingärtner, O.; Möbius-Winkler, S.; Schulze, P.C. Acute myocarditis after COVID-19 vaccination with mRNA-1273 in a patient with former SARS-CoV-2 infection. ESC Heart Fail. 2021, 8, 4710–4714.
134. Tailor, P.D.; Feighery, A.M.; El-Sabawi, B.; Prasad, A. Case report: Acute myocarditis following the second dose of mRNA-1273 SARS-CoV-2 vaccine. Eur. Heart J. Case Rep. 2021, 5, ytab319.
135. Sulemankhil, I.; Abdelrahman, M.; Negi, S.I. Temporal Association between the COVID-19 Ad26.COV2.S Vaccine and Acute Myocarditis: A Case Report and Literature Review. Cardiovasc. Revasc. Med. 2022, 38, 117–123.
136. Kim, I.C.; Kim, H.; Lee, H.J.; Kim, J.Y.; Kim, J.Y. Cardiac Imaging of Acute Myocarditis Following COVID-19 mRNA Vac-cination. J. Korean Med. Sci. 2021, 36, e229.
137. Patrignani, A.; Schicchi, N.; Calcagnoli, F.; Falchetti, E.; Ciampani, N.; Argalia, G.; Mariani, A. Acute myocarditis following Comirnaty vaccination in a healthy man with previous SARS-CoV-2 infection. Radiol. Case Rep. 2021, 16, 3321–3325.
138. Hasnie, A.A.; Hasnie, U.A.; Patel, N.; Aziz, M.U.; Xie, M.; Lloyd, S.G.; Prabhu, S.D. Perimyocarditis following first dose of the mRNA-1273 SARS-CoV-2 (Moderna) vaccine in a healthy young male: A case report. BMC Cardiovasc. Disord. 2021, 21, 375.
139. Isaak, A.; Feisst, A.; Luetkens, J.A. Myocarditis Following COVID-19 Vaccination. Radiology 2021, 301, E378–E379.
140. Williams, C.B.; Choi, J.I.; Hosseini, F.; Roberts, J.; Ramanathan, K.; Ong, K. Acute Myocarditis Following mRNA-1273 SARS-CoV-2 Vaccination. CJC Open 2021, 3, 1410–1412.
141. Nassar, M.; Nso, N.; Gonzalez, C.; Lakhdar, S.; Alshamam, M.; Elshafey, M.; Abdalazeem, Y.; Nyein, A.; Punzalan, B.; Durrance, R.J.; et al. COVID-19 vaccine-induced myocarditis: Case report with literature review. Diabetes Metab. Syndr. 2021, 15, 102205.
142. Cereda, A.; Conca, C.; Barbieri, L.; Ferrante, G.; Tumminello, G.; Lucreziotti, S.; Guazzi, M.; Mafrici, A. Acute myocarditis after the second dose of SARS-CoV-2 vaccine: Serendipity or atypical causal relationship? Anatol. J. Cardiol. 2021, 25, 522–523.
143. Watkins, K.; Griffin, G.; Septaric, K.; Simon, E.L. Myocarditis after BNT162b2 vaccination in a healthy male. Am. J. Emerg Med 2021, 50, 815.e1–815.e2.
144. Deb, A.; Abdelmalek, J.; Iwuji, K.; Nugent, K. Acute Myocardial Injury Following COVID-19 Vaccination: A Case Report and Review of Current Evidence from Vaccine Adverse Events Reporting System Database. J. Prim. Care Community Health 2021, 12, 21501327211029230.

145. Habib, M.B.; Hamamyh, T.; Elyas, A.; Altermanini, M.; Elhassan, M. Acute myocarditis following administration of BNT162b2 vaccine. IDCases 2021, 25, e01197.
146. Minocha, P.K.; Better, D.; Singh, R.K.; Hoque, T. Recurrence of Acute Myocarditis Temporally Associated with Receipt of the mRNA Coronavirus Disease 2019 (COVID-19) Vaccine in a Male Adolescent. J. Pediatr. 2021, 238, 321–323.
147. Muthukumar, A.; Narasimhan, M.; Li, Q.Z.; Mahimainathan, L.; Hitto, I.; Fuda, F.; Batra, K.; Jiang, X.; Zhu, C.; Schoggins, J.; et al. In-Depth Evaluation of a Case of Presumed Myocarditis after the Second Dose of COVID-19 mRNA Vaccine. Circulation 2021, 144, 487–498.
148. McLean, K.; Johnson, T.J. Myopericarditis in a previously healthy adolescent male following COVID-19 vaccination: A case report. Acad. Emerg. Med. 2021, 28, 918–921.
149. Albert, E.; Aurigemma, G.; Saucedo, J.; Gerson, D.S. Myocarditis following COVID-19 vaccination. Radiol. Case Rep. 2021, 16, 2142–2145.
150. Bautista García, J.; Peña Ortega, P.; Bonilla Fernández, J.A.; Cárdenes León, A.; Ramírez Burgos, L.; Caballero Dorta, E. Mio-carditis aguda tras administración de vacuna BNT162b2 contra la COVID-19 [Acute myocarditis after administration of the BNT162b2 vaccine against COVID-19]. Rev. Esp. Cardiol. 2021, 74, 812–814.
